# Genomic prediction of cotton fibre quality and yield traits using Bayesian regression methods

**DOI:** 10.1038/s41437-022-00537-x

**Published:** 2022-05-06

**Authors:** Zitong Li, Shiming Liu, Warren Conaty, Qian-Hao Zhu, Philippe Moncuquet, Warwick Stiller, Iain Wilson

**Affiliations:** 1 CSIRO Agriculture & Food, GPO Box 1600, Canberra, ACT 2601 Australia; 2 CSIRO Agriculture & Food, Locked Bag 59, Narrabri, NSW 2390 Australia

**Keywords:** Plant breeding, Genome

## Abstract

Genomic selection or genomic prediction (GP) has increasingly become an important molecular breeding technology for crop improvement. GP aims to utilise genome-wide marker data to predict genomic breeding value for traits of economic importance. Though GP studies have been widely conducted in various crop species such as wheat and maize, its application in cotton, an essential renewable textile fibre crop, is still significantly underdeveloped. We aim to develop a new GP-based breeding system that can improve the efficiency of our cotton breeding program. This article presents a GP study on cotton fibre quality and yield traits using 1385 breeding lines from the Commonwealth Scientific and Industrial Research Organisation (CSIRO, Australia) cotton breeding program which were genotyped using a high-density SNP chip that generated 12,296 informative SNPs. The aim of this study was twofold: (1) to identify the models and data sources (i.e. genomic and pedigree) that produce the highest prediction accuracies; and (2) to assess the effectiveness of GP as a selection tool in the CSIRO cotton breeding program. The prediction analyses were conducted under various scenarios using different Bayesian predictive models. Results highlighted that the model combining genomic and pedigree information resulted in the best cross validated prediction accuracies: 0.76 for fibre length, 0.65 for fibre strength, and 0.64 for lint yield. Overall, this work represents the largest scale genomic selection studies based on cotton breeding trial data. Prediction accuracies reported in our study indicate the potential of GP as a breeding tool for cotton. The study highlighted the importance of incorporating pedigree and environmental factors in GP models to optimise the prediction performance.

## Introduction

Cotton, primarily the tetraploid species *Gossypium hirsutum*, is widely grown in more than 70 countries. It is the world’s leading renewable textile fibre crop (Paterson et al. 2012), as well as an important source of plant oil and protein (Jabran et al. 2019). The goal of cotton breeding is to achieve genetic gains for desirable fibre characteristics, disease resistance and yield traits in a time and cost-efficient manner. The Commonwealth Scientific and Industrial Research Organisation (CSIRO) cotton breeding program combines traditional field phenotyping and molecular marker assisted selection. Most economically important cotton traits are polygenic in nature making them difficult and expensive to manipulate and improve. As a result, a complete breeding cycle, from initial crossing to commercial release, takes a minimum of eight to ten years (Stiller and Wilson 2014). Genomic selection or genomic prediction (GP) is a relatively new molecular based breeding technology (Meuwissen et al. 2001). It has the potential to improve cotton breeding programs by efficiently allocating resources used in the breeding process by selecting individuals before being tested in field experiments based on genomic estimated breeding value (GEBV), reducing the breeding cycle interval (Crossa et al. 2017; Jannink et al. 2010) and the identification of genotypes that can be used as parents in future crosses to advance specific breeding objectives.

GP (Meuwissen et al. 2001) has been applied as a quantitative molecular breeding tool for crop improvement in various species including, but not limited to, wheat (Poland et al. 2012), maize (Millet et al. 2019), rice (Spindel et al. 2015), and soybean (Jarquín et al. 2014a). Briefly, GP uses a statistical predictive model based on a training population with known genotype and phenotype information to predict genomic breeding value of a related test population with available genotype information. The training population is used to estimate the model parameters, quantifying the association between the phenotypes and genotypes. Once the model is developed, those parameters are used to calculate GEBVs for test population with only known genotype information. Ideally, the prediction accuracy should be evaluated by comparing the GEBVs to true breeding values (TBVs). However, in practice TBVs are not available, thus phenotype scores are used as a surrogate to assess the accuracy of prediction. This evaluation process is important when developing and assessing a GP model.

Compared to conventional phenotype-based breeding approaches, GP may be less costly and more time effective than the traditional phenotype-based breeding approaches. Secondly, GP can be conducted in the early stages of plant development such as on the seed and/or early segregating generations of breeding populations so that a population can be enriched for desired plant characteristics. This process has the potential to reduce the breeding cycle (Crossa et al. 2017), resulting in more efficient use of costly phenotyping resources. Finally, unlike marker assisted selection which utilises a few large effect quantitative trait loci (QTL) for trait improvement, GP utilises genome-wide DNA variation. Therefore, it can capture many small QTL effects, which may cumulatively have a large contribution to the phenotype variation. Hence, GP is more appropriate to predict complex agronomic traits controlled by polygenic gene effects (Goddard and Hayes 2007).

Several factors may impact the power of GP to predict phenotype outcomes. These factors include the heritability and genetic architecture of quantitative traits under evaluation, population structure, the quality of phenotyping and genotyping, the density of markers, the size of the training population, the degree of relatedness between the training and test populations, and the statistical models being used to conduct prediction (Zhang et al. 2019).

Introduction of more data into the training population is usually beneficial for prediction accuracies. However, the inclusion of individuals that are unrelated to the test population in the training set may reduce the prediction accuracy (Wolc et al. 2016; Edwards et al. 2019). Hence, a training set optimisation procedure will improve prediction accuracies (Rincent et al. 2012; Akdemir et al. 2015; Berro et al. 2019). Alternatively, the relatedness between the training and test sets could also be considered by including the relationship among the individuals into the statistical model. Pairwise relationship coefficients can be inferred based on the known pedigree, and the corresponding relationship matrix can be incorporated as a random effect in the statistical predictive model. When pedigree information is lacking, unsupervised clustering approaches (Xu and Tian 2015; Pritchard et al. 2000) can be applied to infer population or family structure, and that information can also be incorporated into the models (Heslot and Jannink 2015; Vandenplas et al. 2018).

GP in cotton breeding is largely still under development as there have been limited GP or pedigree-based studies in cotton and largely, exiting studies have concentrated on fibre quality traits. Gapare et al. (2018) conducted a small-scale GP study on 215 historical varieties collected from the CSIRO breeding program. Cross validation (CV) results revealed that a number of prediction methods including genomic best linear unbiased prediction (Endelman 2011) and Bayesian AlphaBeta methods (Meuwissen et al. 2001) could provide promising prediction accuracies (i.e. up to 0.7 in terms of Pearson correlation between GEBVs and phenotypes) for fibre length and strength. The study also highlighted the importance of taking account of environmental factors in the prediction models. Islam et al. (2020) evaluated similar predictive methods as Gapare et al. (2018) on a multiple parental cross population comprising 550 lines with six fibre quality traits measured using CV. The study also yielded high prediction accuracies up to 0.69 for several fibre quality traits. Liu et al. (2020) proposed an alternative strategy using the sequence variations of 474 fibre length genes and their expression data during fibre development to conduct prediction for fibre length. Using a training population of 128 recombinant inbred lines, the prediction accuracy for fibre length was up to 0.83. Another relevant study by Pérez et al. (2015) used pedigree-based relationship matrix as a basis to predict yield using multiple environmental trials. Although it is not a GP study, it used pedigree data in a genotype or gene-environmental interaction model to achieve prediction accuracy of around 0.5.

The objective of this study is to build on previous cotton GP research to enhance the capability of a cotton GP model using fibre quality properties (fibre length, strength, SFI, elongation, micronaire, and uniformity), lint percentage and lint yield from 1385 cotton breeding lines collected from the CSIRO cotton breeding program. The study hypothesised that: (1) prediction accuracies will be improved through the combination of genomic and pedigree information in cotton GP models. Prediction analyses were conducted using parametric regression methods including Bayesian genomic best linear unbiased predictor (BG-BLUP), Bayesian LASSO and Bayes C, as well as combining these three models with a random effect to account for pedigree. In addition, a non-parametric Bayesian additive regression tree (BART) approach (Chipman et al. 2010; Waldmann 2016) was also applied to our data sets. All the models include year and trial information as covariates to account for the environmental effects. The performance of these models was evaluated in three prediction scenarios. In scenario 1, CV was adopted to randomly divide samples into multiple parts, and then used one part of the data in turn as the test population, and the rest as the training population. The scenario 2 used the latest 2017 data as the test population, and the previous data as the training, to mimic predictions based on unknown genotypes in unknown environments. The scenario 3 considered nine separate biparental families collected in 2017 as the test population, and evaluated whether using the whole training set, or only a subset of the training data that is relevant to the test set could lead to better prediction. This study is important as adopting novel approaches to improve prediction accuracies is essential for deploying GP models in a commercial breeding program. Only once accuracies reach a sufficient level (e.g. equivalent or better than phenotype selection) can an increase in the rate of genetic gain predicted by GP be realised.

## Materials and methods

### Phenotype data and analysis

The phenotype data used in this study included lint yield (LY; kg ha^−1^), lint percent (LP; percentage of lint of seed cotton, %), and the fibre quality parameters of fibre length (LEN; upper half-mean length of sample), uniformity (UNI; the ratio of the mean fibre length to the upper half-mean length, expressed as %), short fibre index (SFI; the proportion by weight of fibre shorter than 12.7 mm), strength (STR; the force required at the breaking point for a bundle of fibres of a given weight and fineness, g tex^−1^), elongation (EL; the extension ability of a bundle of fibres up to its breaking point, expressed as a % increase over its original length), and micronaire (MIC, a measure of air permeability of compressed fibre samples, which is a composite indication of fibre linear density and maturity, unitless).

All phenotype data were collected from experiments conducted under fully irrigated conditions at the CSIRO cotton breeding program’s core research base at the Australian Cotton Research Institute (ACRI, 30° 12’S, 149°36’E) located at Myall Vale, Narrabri NSW, Australia. The climate at Myall Vale is semi-arid, characterised by mild winters, hot summers and summer-dominant rainfall patterns, with an annual average precipitation of 646 mm (Aust. BOM 2018). The soil of the site is a uniform grey cracking clay (USDA soil taxonomy: Typic Haplustert; Australian soil taxonomy: Grey Vertosol). Plant available soil water to 1.2 m at the site is between 160 and 180 mm (Tennakoon and Hulugalle 2006).

Experiments were laid out in row-column designs with four replicates, generated from CycDesigN software (VSN International, Hemel Hempstead, UK). Each plot consisted of three 10–12 m rows of cotton (depending on the individual experiment). A row spacing of 1 m was used with a planting density of about 10–12 plants m^−2^. Management for all field experiments followed then or current high-input commercial practices, e.g. fully irrigated conditions with careful weed and insect control. Plots were furrow irrigated every 10–14 days (approximately 1 ML ha^−1^ applied at each irrigation) from December through to March, according to crop requirements. Each experiment was managed according to its individual requirements for irrigation, weed and pest control, with all plots receiving the same management regime. At approximately 60% open bolls, crops were defoliated with thidiazuron, and mature un-opened bolls were opened with ethephon. A second application of thidiazuron and ethephon was applied 7–10 days later.

Phenotype data were collected from 1385 lines across 42 experiments conducted between 1993 and 2017 (Table 1). Lines were predominantly at the F_4_ to F_6_ generation, depending on the initial self-generation of breeding families used for single plant selection and then derived breeding lines are tested in the stage-by-stage performance test trials (Liu and Constable 2017). The lines were both conventional and genetically modified (GM); including a mix of released cultivars as well as breeding lines undergoing different stage performance testing. Most of the 1385 lines (~85%) were phenotyped post-2014, and 215 lines included previously published phenotype data (Gapare et al. 2018). Note that some of the 215 lines collected pre-2014 were phenotyped in multiple years and experiments, and all the lines collected in or after 2014 were only phenotyped once. In total, this results in 1907 phenotype observations.

**Table 1:** Details of the lines studied.

Year	No. Lines		No. Biparental crosses	No. Experiments	Notes
	Conventional	Transgenic			
1993/2013	215	–	87	21	37 released cultivars, 178 breeding lines
2014/2015	171	–	11	3	Preliminary to advanced stage material
2015/2016	244	145^a^	37	7	Preliminary to advanced stage material
2016/2017	216	60^b^	15	5	Preliminary to advanced stage material
2017/2018	274	60^b^	17	6	Preliminary to advanced stage material

^a^Transgenic lines with B3F traits.

^b^Transgenic lines with B3XFlex traits.

At harvest, seed cotton was mechanically harvested from the middle row of each plot with a spindle picker (modified Case International 1822) and weighed. The outside rows were not harvested and acted as buffers to minimise the edge effect and inter-plot competition. LP was determined from a 300 g sub-sample of the seed cotton that was ginned in a 20 saw gin with a pre-cleaner (Continental Eagle, Prattville, AL U.S.A.), and was subsequently used to calculate lint yield (kg ha^−1^). Lint samples were collected and tested for fibre quality using a Spinlab High Volume Instrument (HVI) model 1000 (Uster Technologies AG, Uster, Switzerland).

Individual experiment’s phenotype data were analysed using linear mixed models taking account of dimensional spatial variation, see Liu et al. (2015) for details. Briefly, the model is described as [Eq. 1]:

1 $$y = {{{\mathrm{X}}}}\tau + {{{\mathrm{Z}}}}_{{{\mathrm{b}}}}\beta _b + {{{\mathrm{Z}}}}_{{{\mathrm{r}}}}\beta _r + {{{\mathrm{Z}}}}_{{{\mathrm{c}}}}\beta _c + \epsilon ,$$

where ***y*** is a vector of plot observation and *τ* represents a vector of fixed genotypic (i.e. line) effects, ***β***_*b*_ is a vector of random effects of replicates (i.e. complete block), ***X***_*τ*_ and ***Z***_*b*_ are the corresponding design matrices. ***β***_*r*_ and ***β***_*c*_ are the vectors of random effects for rows and columns of the experiment with their corresponding design matrices of ***Z***_*r*_ and ***Z***_*c*_. Finally, ***ε*** is a vector of plot errors. Plot errors in the model are assumed to be autocorrelated along experiment dimensions, i.e. row and column, and modelled by the first order separable autoregressive process (AR1) covariance model. The best linear unbiased estimates of test lines from individual trial analysis were pooled together and used as the phenotype value in the GP analyses.

### Genotype data

DNA isolation and SNP genotyping and calling were performed as per Gapare et al. (2018). Leaves from 10–12 plants from each line were combined for DNA extraction using the DNeasy PlantMini Kit (Qiagen) according to the manufacturer’s instructions. All DNA samples were quantified using a NanoDrop 1000 (Thermo Scientific) and normalised to the same concentration (Zhu et al. 2016). DNA at 50 ng/µL for each of lines was processed according to Illumina protocols and hybridised to the CottonSNP63K array at CSIRO Agriculture and Food (Brisbane, Australia) according to the manufacturer’s instructions. Chips were scanned using the Illumina iScan and analysed using the GenomeStudio Genotyping Module (v2.0, Illumina). Genotype calls for each SNP were performed based on the cluster file generated specifically for the CottonSNP63K array (Hulse-Kemp et al. 2015). The SNP calling was performed as for a diploid species so at each locus there are three possible genotypes—AA, AB, and BB. Filtering was performed to return polymorphic SNPs with call rate above 85% and minor allele frequency higher than 2.5%. A set of 12296 polymorphic SNPs were used for model training and GPs. These SNPs were distributed across all the 26 chromosomes of cotton with a density of 6 SNPs/Mbp. The missing genotype values at each marker were imputed based on known genotypes at its flanking markers using a Hidden Markov Chain model (Browning and Browning 2007), implemented in the R package “Synbreed” (Wimmer et al. 2012). Principal component analysis (PCA) was conducted on the genotype data using our own R code to investigate the genetic diversity among the samples.

### The BG-BLUP model

The linear mixed effect (LMM) model can be specified as [Eqs. 2–4]

2 $$y_i = \beta _0 + u_i + W\alpha + Z\gamma + e_i,$$

where *y*_*i*_ is the phenotype record of the *i*th individual (*i* = 1,…, *n*; *n* is the total number of individuals), *β*_0_ is the model intercept, *e*_*i*_ is the residual error: $e = \left[ {e_1, \ldots ,e_n} \right]\sim N\left( {0,{{{\mathrm{I}}}}\sigma _e^2} \right)$ (mutually independent for *i* = 1,…,*n*), $\sigma _e^2$ is the residual variance, *u*_*i*_ is the random effect of SNP markers which follows a normal distribution Eq. 3

3 $${{{\mathbf{u}}}} = \left[ {u_1, \ldots ,u_n} \right]\sim N\left( {0,\sigma _g^2{{{\mathrm{G}}}}} \right),$$

where $\sigma _g^2$ is the additive genetic variance, **G** is the genomic relationship matrix (GRM) Eq. 4 (referred as Method 1 in Van Raden 2008) estimated by

4 $$G_{ik} = \frac{1}{p}\mathop {\sum}\limits_{j = 1}^p {\frac{{\left( {x_{ij} - 2p_j} \right)\left( {x_{kj} - 2p_j} \right)}}{{2p_j\left( {1 - p_j} \right)}},}$$

where *x*_*ij*_ is the genotype value of the *i*th individual and *j*th SNP, coded as −1, 0, and 1 for the three genotypes AA, AB and BB, respectively, *p*_*j*_ represents the minor allele frequency of the *j*th marker. In the Eq. (2), W and Z are the design matrices of experiments and years (Table S1) from which those individuals are collected, and *α* and *γ* are the corresponding random effects of experiments and years, with both following normal distributions: $\alpha \sim N\left( {0,{{{\mathrm{I}}}}\sigma _\alpha ^2} \right)$ and $\gamma \sim N( {0,{{{\mathrm{I}}}}\sigma _\gamma ^2} )$.

In Frequentist statistics, the model parameters including the variance components $\sigma _0^2$ and $\sigma _g^2$ could be estimated by the restricted maximum likelihood algorithm (Harville 1977). Alternatively, the LMM could be formulated into a Bayesian posterior model as [Eq. 5–6]

5 $$\begin{array}{l}P\left( {\beta _0,{{{\mathrm{u}}}},\alpha ,\,\gamma ,\sigma _e^2\left| y \right.} \right) = P\left( {\left. {{{\boldsymbol{y}}}} \right|\beta _0,{{{\mathrm{u}}}},\alpha ,\gamma ,\sigma _e^2} \right)P\left( {\beta _0} \right)\\ \qquad\qquad\qquad\qquad\qquad \times P\left( {{{\mathrm{u}}}} \right)P\left( \alpha \right)P\left( \gamma \right)P\left( {\sigma _e^2} \right)\end{array}$$

where $p\left( {\left. {{{\boldsymbol{y}}}} \right|\beta ,\sigma _0^2} \right)$ is the likelihood, specifying the Eq. (2) in the probability form:

6 $$P\left( {\left. {{{\boldsymbol{y}}}} \right|\beta _0,{{{\mathrm{u}}}},\alpha ,\gamma ,\sigma _e^2} \right) = \mathop {\prod}\limits_{i = 1}^n {\frac{1}{{\sqrt {2\pi \alpha _0^2} }}{{{\mathrm{exp}}}}\left( { - \frac{{\left( {y_i - \beta _0 - u_i - W\alpha - Z\gamma } \right)^2}}{{2\sigma _e^2}}} \right)} ,$$

and $P\left( {\beta _0,{{{\mathrm{u}}}},\sigma _e^2} \right) = P\left( {\beta _0} \right)P\left( {{{\mathrm{u}}}} \right){{{\mathrm{P}}}}\left( {\sigma _e^2} \right)P\left( \alpha \right)P\left( \gamma \right)$ is the prior distribution of the parameters. The prior of the random effect **u**: *P*(**u**) is as specified in (3), where the variance component $\sigma _g^2$ together with the residual variance $\sigma _e^2$ are further assigned with Scaled inverse chi-squared distribution hyper priors, as suggested in Pérez and de los Campos (2014, File S1). Briefly, the degree of freedom (df) parameter is set to be 5, and the scale parameter is specified as *S*_0_ = var(y) × (1 − *R*^2^) × (df + 2) and *S*_*g*_ = var(y) × *R*^2^ × (df + 2)/mean(diag(G)) for $\sigma _e^2$ and $\sigma _g^2$, respectively, where *R*^2^ is specified as 0.5. These settings correspond to the assumption that 50% of the phenotype variance is explained by the genomic variance component, which is suggested by Pérez and de los Campos (2014). Based on our experiment (results not shown), using alternative *R*^2^ values would not lead to any drastic change in heritability estimation and the GP results.

On the basis of the variance components estimated from the Eq. (2), the genomic heritability of a trait can be calculated

$$h^2 = \frac{{\widehat {\sigma _g^2}}}{{\widehat {\sigma _g^2} + \widehat {\sigma _e^2} + \widehat {\sigma _\alpha ^2} + \widehat {\sigma _\gamma ^2}}}$$

This is a Bayesian alternative to the popular GCTA approach (Yang et al. 2011) for estimating genomic heritability of quantitative traits.

### The BG-BLUP combined with pedigree

To incorporate the pedigree information, the BG-BLUP model (2) can be extended as [Eq. 7–8]

7 $$y_i = \beta _0 + u_i + v_i + W\alpha + Z\gamma + e_i,$$

where all terms are defined in the same way as in Eq. (2), except that *v*_*i*_ is a newly added random effect that has the covariance structure representing the pedigree-based relationship matrix **A**, so that the prior of *v*_*i*_ is specified as

8 $$P\left( {{{\mathbf{v}}}} \right)\sim N\left( {0,\,\sigma _a^2A} \right)$$

The pedigree-based matrix **A** was calculated based on the genealogical information of the lines in the study tracing back to five generations (Fig. S1), using the R package “pedigree” (Coster 2015). Like the variance component $\sigma _g^2$, the variance component $\sigma _a^2$ can also be assigned with a scaled inverse chi-squared distribution prior (Pérez and de los Campos 2014). Now, the scale parameters for variance components are $\sigma _e^2$, $\sigma _g^2$ and $\sigma _a^2$ that are specified as $S_0 = {{{\mathrm{var}}}}\left( {{{\mathrm{y}}}} \right) \times \left( {1 - R_a^2 - R_g^2} \right) \times \left( {{{{\mathrm{df}}}} + 2} \right)$, $S_g = {{{\mathrm{var}}}}\left( {{{\mathrm{y}}}} \right) \times R_g^2 \times \left( {{{{\mathrm{df}}}} + 2} \right)/{{{\mathrm{mean}}}}\left( {{{{\mathrm{diag}}}}\left( {{{\mathrm{G}}}} \right)} \right)$ and $S_a = {{{\mathrm{var}}}}\left( {{{\mathrm{y}}}} \right) \times R_a^2 \times \left( {{{{\mathrm{df}}}} + 2} \right)/{{{\mathrm{mean}}}}\left( {{{{\mathrm{diag}}}}\left( {{{\mathrm{A}}}} \right)} \right)$, respectively, where $R_g^2$ = 0.4 and $R_g^2$ = 0.1, corresponding to the model assumption that 40% of phenotype variance explained by genetics, and 10% explained by the pedigree information, based on the assumption that the genetic relationship would capture more phenotypic variation than the pedigree relationship (Fraimout et al. 2021). In addition, based on our numerical experiment (results not shown), changing those prior values would not lead to drastic change in the prediction results.

### Bayesian LASSO

Additional to the BG-BLUP model building the relation between phenotypes and GRM, a multiple locus model could also be applied directly to evaluate the association between phenotypes and different markers as

$$y_i = \beta _0 + \mathop {\sum}\limits_j^p {x_{ij}\beta _j} + W\alpha + Z\gamma + e_i,$$

or alternatively, in a likelihood form [Eq. 9]:

9 $$P\left( {\left. y \right|\beta ,\sigma _e^2} \right) = \mathop {\prod}\limits_{i = 1}^n {\frac{1}{{\sqrt {2\pi \sigma _e^2} }}} {{{\mathrm{exp}}}}\left( { - \frac{{\left( {y_i - \beta _0 - \mathop {\sum}\nolimits_{j = 1}^p {x_{ij}\beta _j - W\alpha - Z\gamma } } \right)^2}}{{2\sigma _e^2}}} \right),$$

where *β*_*j*_ (*j* = 1,…, *p*) is the regression coefficient representing the additive genetic effect of the marker *j*, and the other symbols including *y*_*i*_, *x*_*ij*_, *β*_*0*_ and $\sigma _e^2$ are defined the same way as in Eq. (2). Given the fact that the number of SNPs is larger (i.e. p > n), it is essential to keep only the SNPs with non-negligible effects, and to exclude SNPs with small effects out of the model. In Bayesian statistics, this can be achieved by using shrinkage prior λ on regression parameters (O’Hara and Sillanpää 2009).

The regression parameter *β*_*j*_ (*j*=1,…, *p*) is the additive genetic effect of marker *j*, which is assumed to follow a double exponential (DE) prior distribution [Eq. 10]:

10 $$P\left( {\beta _j} \right) = \lambda {{{\mathrm{exp}}}}\left( { - \lambda \left| {\beta _j} \right|} \right)$$

The DE distribution has a heavier tail than the normal distribution, and it can shrink the effects of unimportant markers towards zero. The parameter λ determines the degree of shrinkage, i.e. how many markers will be excluded from the model. The DE distribution could be further represented as a scale mixture distribution of a normal distribution of *β* and an exponential distribution, which inspires the following hierarchical prior setting of the regression parameter *β*_*j*_ in Bayesian LASSO [Eq. 11–12]:

11 $$P\left( {\beta _j} \right) = N\left( {\beta _j\left| 0 \right.,\sigma _j^2} \right),$$

12 $$P\left( {\sigma _j^2} \right) = {{{\mathrm{Exp}}}}\left( {\sigma _j^2\left| {\frac{{\lambda ^2}}{2}} \right.} \right)$$

The shrinkage factor λ^2^ is further assigned with a hyper prior of a Gamma distribution Gamma (λ^2^|s, r), and so it can be estimated as other model parameters. The shape and rate parameters of the Gamma prior was specified to s = 1.1 and $r = \frac{{\left( {s - 1} \right)}}{{2 \times \left( {1 - R^2} \right)/R^2 \times MSx}}$ (Pérez and de los Campos 2014), where MSx represents the sum of the variances of genotype values of each SNP, and *R*^2^ = 0.5.

### Bayes C

Another popular way to achieve shrinkage estimation is to assign a spike and slab prior (Ishwaran and Rao 2005) to the regression parameters as follows [Eq. 13]:

13 $$P\left( {\beta _j\left| {\lambda _j} \right.} \right) \propto \left( {1 - \gamma _j} \right){{{\mathrm{I}}}}_{\left( {\beta _{{{\mathrm{j}}}} = 0} \right)} + \gamma _{{{\mathrm{j}}}}{{{\mathrm{N}}}}\left( {\left. {\beta _{{{\mathrm{j}}}}} \right|0,\sigma _{{{\mathrm{b}}}}^2} \right),$$

where γ_*j*_ is a binary indicator variable to tell whether the genetic effect of SNP *j* should be non-negligible and follow a normal distribution, or whether the effect is small and assigned with a zero value. In formula (13), the indicator variable γ_*j*_ and the variance component $\sigma _{{{\mathrm{b}}}}^2$ are further assigned with priors of Bernoulli: *Bern*(γ_*j*_|*π*) and Inverse chi-squared:$IG\left( {\sigma _{{{\mathrm{b}}}}^2\left| {df} \right.,S_0} \right)$, respectively. In the Inverse gamma prior $IG\left( {\sigma _{{{\mathrm{b}}}}^2\left| {df} \right.,S_0} \right)$, the parameters df = 5 and *S*_0_ = var(*y*) × *R*^2^ × (*df* + 2)/*MSx*, with *R*^2^ = 0.5. In the Bernoulli prior Bern(γ_*j*_|π), the parameter π was further assigned with a Beta prior Beta(π | p_0_, π_0_), with p_0_ = 50, and π_0_ = 0.5_._ The spike and slab prior (13) is often referred as the Bayes C model (Habier et al. 2011) in the GP literature.

### Adding pedigree into the Bayesian LASSO and Bayes C model

To account for the pedigree information, a random effect **u** could be further added into the likelihood (9) as [Eq. 14]

14 $$P\left( {\left. {\bf{y}} \right|\boldsymbol{\beta} ,\sigma _e^2} \right) = \mathop {\prod}\limits_{i = 1}^n {\frac{1}{{\sqrt {2\pi \sigma _0^2} }}{{{\mathrm{exp}}}}\left( { - \frac{{\left( {y_i - \beta _0 - \mathop {\sum}\nolimits_{j = 1}^p {x_{ij}\beta _j - W\alpha - Z\gamma - u_i} } \right)^2}}{{2\sigma _e^2}}} \right)} ,$$

where the random effect **u** following a normal prior as:

$${{{\boldsymbol{u}}}}\sim N\left( {0,\sigma _a^2{{{\mathbf{A}}}}} \right),$$

where A is the pedigree-based relationship matrix. The prior information for all other parameters in (14) could be assigned in the exact same way as in Bayesian LASSO or Bayes C.

Thus, both the Bayesian LASSO (or Bayes C, which has a different prior setting for marker effects) and BG-BLUP model utilise the same covariance matrix **A** constructed from the pedigree analysis, to account for the family structure. However, the two model classes use different way to model the dependency between genotype and phenotype data. Bayesian LASSO or Bayes C used a multiple locus model with a shrinkage prior to estimate the additive effects of SNPs. The BG-BLUP model used the SNP data to estimate the GRM to study the genotype-phenotype association.

The posterior distribution of all the three models can be evaluated using the Markov Chain Monte Carlo (MCMC) algorithm, in particular the Gibbs sampling, as presented in de los Campos et al. (2009) and Pérez and de los Campos. (2014). Practically, the MCMC was implemented by the R package BGLR (Pérez and de los Campos. 2014). The algorithm generated 50,000 posterior samples, with the first 10,000 samples as burn-in, and every 20th of the rest were stored to reduce the serial correlation.

### Bayesian additive regression tree

Bayesian additive regression tree (BART) (Chipman et al. 2010; Hill et al. 2020) is a more flexible model structure compared to the linear regression, presented as [Eq. 15]

15 $${{{\mathrm{y}}}}_{{{\mathrm{i}}}} = {{{\mathrm{f}}}}\left( {{{{\mathrm{x}}}}_{{{\mathrm{i}}}}} \right) + e_i,$$

where f represents a summation of many regression trees as

$$f\left( x \right) = \mathop {\sum}\limits_{k = 1}^m {g\left( {x,T_k,M_k} \right),}$$

where *T*_*k*_ represents a binary decision tree with a set of terminal nodes and interior decision rules, $\mu _{{{{\mathrm{kl}}}}} \in M_k\left( {{{{\mathrm{l}}}} = 1, \ldots ,b_k} \right)$ are a set of parameter values associated with each terminal nodes of *T*_*k*_, and *e*_i_ is the Gaussian residual error as in (2).The decision rules associated with *T*_*k*_ are binary splits of the genotypes **x** to $\left( {{{{\boldsymbol{x}}}} \in A_{kl}} \right)$ and $\left( {{{{\boldsymbol{x}}}} \notin A_{kl}} \right)$, where A is a subset of **x**. The function g then assigns each *μ*_*kl*_ to **x** as

$$g\left( {{{{\boldsymbol{x}}}},T_k,M_k} \right) = \mu _{kl}\,{{{\mathrm{if}}}}\,{{{\boldsymbol{x}}}} \in A_{k{{{\mathrm{l}}}}}$$

In BART, a regularised prior is assigned to each tree *T*_k_ and its terminal notes *M*_k_, and assumed independence between different components:

$$\begin{array}{lll}p\left( {\left( {T_1,M_1} \right),\left( {T_2,M_2} \right), \ldots ,\left( {T_m,M_m} \right)} \right) &=& \mathop {\prod}\limits_{k = 1}^m {p\left( {T_k,M_k} \right)} \\ &=& \mathop {\prod}\limits_{k = 1}^m {p\left( {T_k} \right)p\left( {M_k{{{\mathrm{|}}}}T_k} \right)} \\& =& \mathop {\prod}\limits_{k = 1}^m {p\left( {T_k} \right)\mathop {\prod}\nolimits_l^{b_k} {\left( {\mu _{kl}\left| {T_k} \right.} \right),} } \end{array}$$

where the prior p(*T*_*j*_) consists of three parts: (i) the probability that a node at the depth *d* is non-terminal given by α(1+*d*)^−β^ where **α** = 0.95, and β = 2 as suggested in Chipman et al. (2010) and (ii) a uniform distribution specified for the variables *x*_*ij*_ (j=1,…,*p*) which are assigned at each interior node for splitting, and (iii) a uniform distribution on the splitting rule assignment in each interior node. The *p*(μ_*kl*_|*T*_*k*_) is a normal distribution $N( {0,\sigma _\mu ^2} )$, with the variance fixed to be $\sigma _\mu ^2 = \frac{{0.5}}{{k\sqrt M }}$, and k = 2, and the number of trees M is fixed to be 200 as Waldmann (2016).

The prior tends to generate a lot of small trees with simple structure, and therefore can avoid the over-fitting problem. Compared to the G-BLUP, Bayes LASSO or Bayes C methods, the benefit of BART is that it can implicitly model not only the additive effects, but also the non-additive genetic effects such as dominance effects and gene-gene interaction effects (Waldmann 2016).

The BART model could be evaluated using Gibbs sampling with a few Metropolis Hasting sampling steps, which can be implemented using the R package BayesTree (Chipman and McCulloch 2016). Here we generated equivalent amount of MCMC samples as for the other three models.

### Assessment of prediction accuracy

Three different scenarios were considered to evaluate the prediction accuracy of different approaches. *In Scenario 1*, a fivefold-CV and an additional 50-fold-CV were used by randomly dividing the samples into multiple parts (i.e. either 5 or 50) with equivalent sizes, and in turn using each part of the data (of 277 lines) as the test population, and the rest (of 1108 lines) as the training data. In fivefold-CV, the training and test set comprise 1108 and 277 lines, respectively. And in 50-fold-CV, the training and test sets comprise 1357 and 28 lines, respectively. In CV, the average predictive performance over different folds is considered as prediction accuracy. *In Scenario 2*, all the lines phenotyped at seasons 1993–2016 were used as the training population to build the model. The lines phenotyped in the most recent (2017/2018) season were used as the test population to calculate the prediction accuracy. This scenario reflects a likely approach of utilising GP in a breeding program where samples collected in previous years are used as the training data to generate GEBVs from the most recent season. *Scenario 3* used the same training population as in Scenario 1, but it focused on predicting separately on each single biparental family collected at 2017/2018 (Schopp et al.^ 2017^; Brauner et al. 2020). For the training data set, we considered either using the whole set of samples up to 2016/2017, or only a subset of samples that are closely relevant to each test population defining by the relationship coefficients between samples calculated based on pedigree. We used relationship coefficients thresholds of both 0.125 and 0.25, representing the first cousin (i.e. sharing one grandparent) and half-sibling relations (i.e. sharing one parent), respectively. This approach explored whether an appropriate training population existed for single biparental family in terms of attaining a better prediction accuracy.

In all these three scenarios, the Pearson correlation coefficient between the GEBV and the phenotypes was considered as the measurement of prediction accuracy.

## Results

In this study, we conducted GP analyses on a total of 1385 lines using eight statistical methods. The methods were BG-BLUP, Bayesian LASSO, Bayes C and a non-parametric BART, used either only genomic data or combined genomic data with pedigree information. The prediction accuracies were then evaluated in three different scenarios.

### Phenotype and genomic variation

A summary of phenotype variation including mean, standard error, minimum value, maximum value of phenotypes for each trait was given in Table 2. Genomic heritabilities estimated by BG-BLUP were 0.59 for LEN, 0.35 for UNI, 0.30 for SFI, 0.59 for STR, 0.46 for EL, 0.42 for MIC, 0.26 for LY, and 0.41 for LP (Table 2).

**Table 2:** Summary of phenotype variation across traits including the mean, standard error, minimum value, and maximum value of original phenotypes and the adjusted phenotypes by checks in each trial to reduce the phenotype variation caused by management and environment.

	LEN	UNI	SFI	STR	EL	MIC	LY	LP
Mean	1.23	84.2	5.54	31.4	14.6	5.2	2683	49.3
SD	0.05	1.21	1.95	1.73	1.74	0.36	525	2.35
Min	1.10	80.6	2.3	26.3	0.9	4.25	1326	32.4
max	1.41	88.5	9.9	49.6	14.6	5.2	3693	49.3
Mean_adjust	1.03	1.00	0.94	1.01	0.96	0.99	1.00	0.99
SD_adjust	0.04	0.01	0.16	0.05	0.10	0.06	0.12	0.04
Min_adjust	0.93	0.96	0.35	0.88	0.35	0.70	0.44	0.76
Max_adjust	1.20	1.05	1.7	1.69	1.38	1.17	1.63	1.12
Genomic H^2^	0.59	0.35	0.30	0.59	0.46	0.42	0.26	0.41
95% Credible intervals for H^2^	(0.53, 0.66)	(0.28, 0.41)	(0.22, 0.37)	(0.53, 0.65)	(0.39, 0.53)	(0.35, 0.49)	(0.19, 0.33)	(0.29, 0.52)

Principal component analysis (PCA) results (Fig. S2) of genomic data revealed no clear separation between the samples collected in different years.

### Prediction scenario 1 (Cross validation)

**Fig. 1:**
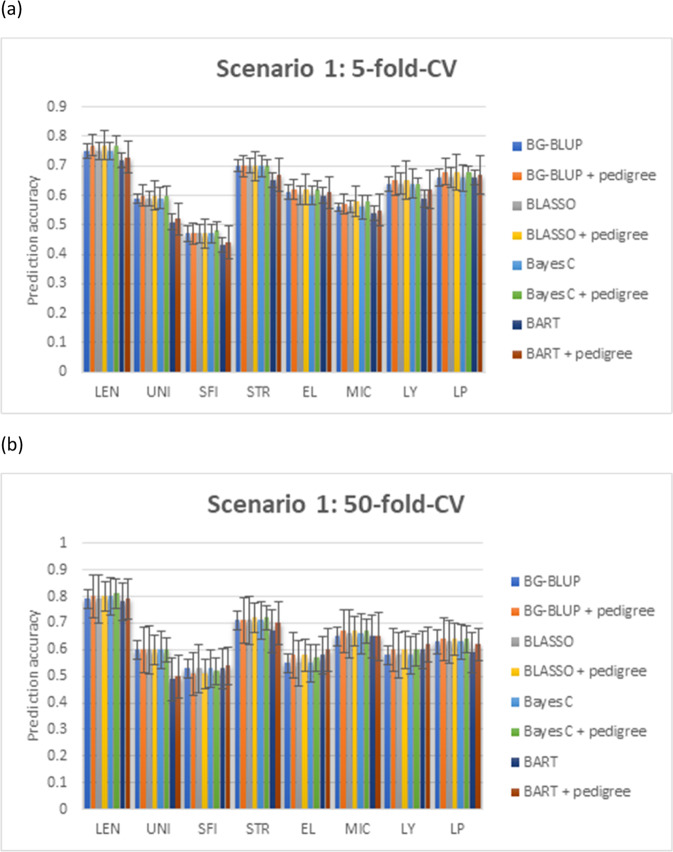
The prediction accuracies and standard errors of scenario 1. (**a** Fivefold cross validation; and **b** 50-fold cross validation). Methods under evaluation were Bayesian genomic best linear unbiased predictor (BG-BLUP), Bayesian LASSO, Bayes C, Bayesian additive regression tree (BART), and these four models further adding pedigree or structure information as random effects. Traits being analysed included fibre length (LEN), uniformity (UNI), short fibre index (SFI), fibre strength (STR), fibre elongation (EL), fibre micronaire (MIC), lint yield (LY) and lint percentage (LP).

When using the fivefold-CV to evaluate the predictability of the models, the accuracies were 0.72–0.77 for LEN, 0.47–0.60 for UNI, 0.43–0.48 for SFI, 0.65–0.71 for STR, 0.60–0.62 for EL, 0.50–0.58 for MIC, 0.59–0.65 for LY, and 0.66–0.68 for LP (Fig. 1a; Table S2). The performance of BG-BLUP, Bayesian LASSO, Bayes C was comparable to each other. BART’s performance was worse than the other three methods for UNI, but was comparable to other traits. Inclusion of pedigree information in all the methods improved the prediction accuracies by 1–3% for different traits (Table S2).

The 50-fold-CV’s accuracies were 0.78–0.80 for LEN, 0.49–0.60 for UNI, 0.51–0.53 for SFI, 0.67–0.71 for STR, 0.55–0.60 for EL, 0.65–0.67 for MIC, 0.58–0.6 for LY, and 0.59–0.64 for LP. Similar as in the fivefold-CV, the inclusion of pedigree in all the Bayesian approaches slightly improved the prediction accuracies (Fig. 1b; Table S3).

### Prediction scenario 2

**Fig. 2:**
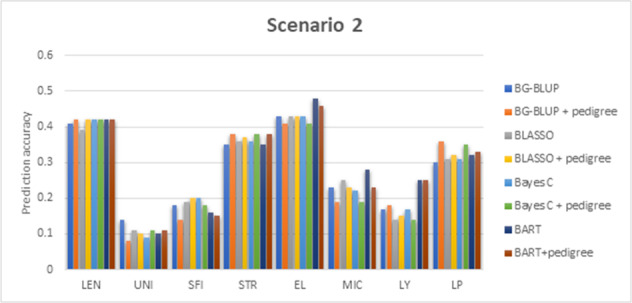
The prediction accuracies of the scenario 2: the lines phenotyped at seasons 1993–2016 were used as the training population, and the data collected in the 2017/2018 season were used as the test population. Methods under evaluation were Bayesian G-BLUP, Bayesian LASSO, Bayes C, BART, and these three models further adding pedigree or structure information as random effects, and BART. Traits being analysed included fibre length (LEN), uniformity (UNI), short fibre index (SFI), fibre strength (STR), fibre elongation (EL), fibre micronaire (MIC), lint yield (LY) and lint percentage (LP).

By using all samples sourced prior to the 2017/18 (1051 lines) as the training population to build the model and the 2017/18 season samples (334 new lines) to evaluate the prediction, the prediction accuracies of the eight methods ranged 0.39–0.42 for LEN, 0.08–0.14 for UNI, 0.14–0.20 for SFI, 0.35–0.38 for STR, 0.41–0.48 for EL, 0.19–0.28 for MIC, 0.14–0.25 for LY, 0.30–0.36 for LP (Fig. 2; Table S4). BG-BLUP and Bayesian LASSO, Bayes C and BART achieved almost identical accuracies for LEN, STR and LP (Table S4), though they are formulated under different model assumptions. The BART method gave higher prediction accuracies for EL, MIC and LY. Bayesian BLUP performed better for UNI. Bayes LASSO performed better for SFI. Adding pedigree information as a random effect to BG-BLUP, Bayesian LASSO, Bayes C or BART, resulted in the highest accuracies for SFI, STR and LP, but had compromised the accuracies of EL and MIC prediction; however, all these changes were small in magnitude (Table S4).

### Prediction scenario 3

Here nine biparental families collected from the year 2017/18 were treated as separate test populations to evaluate whether using the families which are closely related to the test population in the training population could improve the prediction.

**Fig. 3:**
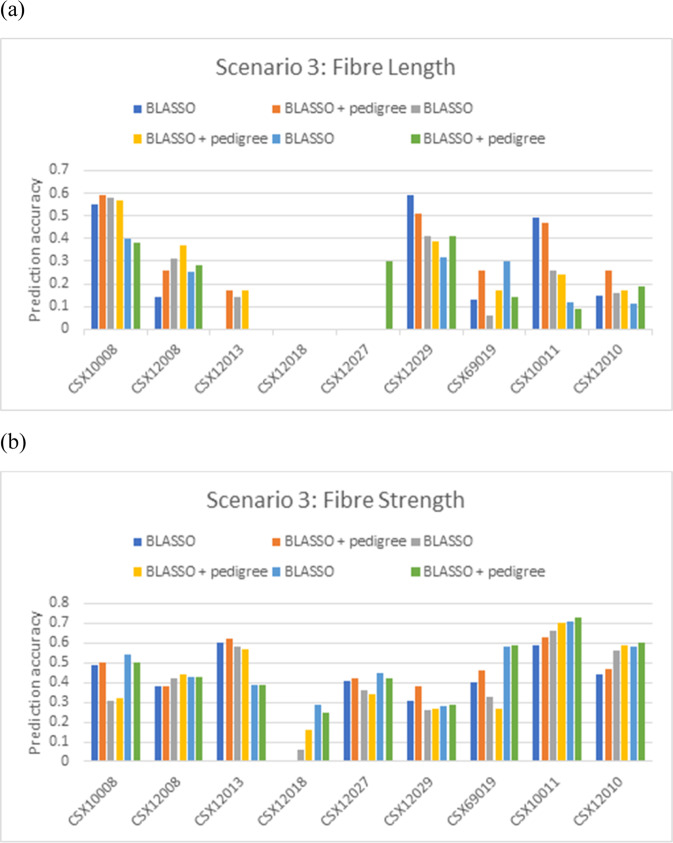
The prediction accuracies of the scenario 3. **a** fibre length (LEN), and **b** strength (STR). This approach used each biparental family from season 2017/2018 as the separate test population. The training population was either all the lines phenotyped before 2017, or the families phenotyped before 2017 which are closely relevant to the target population (i.e. the related coefficient no < 0.125 or 0.25).

The prediction accuracies for the Bayesian LASSO based on the whole training population (average over the 9 biparental families) were 0.23 for LEN, 0.25 for UNI, 0.14 for SFI, 0.4 for STR, 0.3 for EL, 0.22 for MIC, 0.13 for LY, and 0.38 for LP (Fig. 3; Table S5). Adding pedigree to Bayesian LASSO produced better prediction accuracies for all of the traits except EL. Using only the families that have relationship coefficients at least 0.125 (i.e. equivalent as sharing one common grandparent) with the test population in the training population yielded best prediction accuracies for MIC and LP (Table S5). Using the families that have relationship coefficients at least 0.25 (i.e. as sharing one parent) produced best prediction accuracies for UNI, SFI, STR, and LY. The whole training data set performed best for LEN and EL.

## Discussion

In this paper, we presented a GP study on both fibre qualities and yield traits of cotton (*G. hirsutum*) with 1385 samples collected from the CSIRO cotton breeding program. This study is on a much larger scale than the previous two GP studies on the same species (Gapare et al. 2018; Islam et al. 2020). From a statistical modelling perspective, we focused on evaluating several Bayesian regression methods including BG-BLUP, Bayesian LASSO, Bayes C, and BART. In all the methods, year and experiment information were added as covariates to account for the environmental effects. The former three methods have already been widely used in the GP literature (Crossa et al. 2017; Wang et al. 2018), while the BART approach is less well known in the field (Waldmann 2016). Since the trait data were collected from multiple biparental families, we calculated the relationship matrix based on pedigree information, and used that matrix in conjunction with the genomic data in the model and observed an improvement in prediction accuracies.

### Prediction accuracies over three scenarios

Scenario 1 aims to evaluate the predictive ability of different predictive approaches by using cross-validation (CV), which are also used in many other GP studies (Runcie and Cheng 2019). In contrast, Scenario 2 reflects a likely approach when utilising GP in a breeding program, where samples collected in the past are used as the training population to generate GEBVs for the new samples from the most recent season. The prediction accuracies for traits in Scenario 1 were significantly higher than those in Scenario 2, which can be explained by how the training and test population being defined. In Scenario 2, the training and test data comprised samples from different biparental families generated in different years, and additionally their phenotypes were subject to different environments (e.g. climate variability across testing seasons). But in Scenario 1, the training and test populations were randomly defined so both have samples collected from the same families and phenotypes measured at the same environments, resulting in more accurate predictions. Our results are aligned with results from existing literature shown that the prediction accuracies of new genotypes in new environments (i.e. Scenario 2 were considerably lower than the prediction by using CV (i.e. randomly defining the training and test population, i.e. Scenario 1) (Jarquín et al. 2014b; Gillberg et al. 2019). Another observation was that the prediction accuracies in Scenarios 1 and 2 were both highly correlated with the square root of the genomic heritabilities across traits. For example, the Pearson correlation coefficients between prediction accuracies by GB-BLUP in Scenario 1 (50-fold-CV) and Scenario 2 and the squared root of heritabilities are 0.80 and 0.82, respectively. Note that the square root of the heritability of a trait was considered as the theoretically expected value of the prediction accuracies could be achieved by GP (Estaghvirou et al. 2013).

The Scenario 3 is related to Scenario 2 but with a focus on calculating GEBVs for within family samples instead of between family samples. Results highlight that the prediction accuracies vary dramatically across different biparental families. This may reflect the complexity of the relationship structure among those families. The pedigree-based relationship matrix had two roles here. First, it was used to tune the training population to select training samples that are closely relevant to the target family. Second, it was also used as a random effect in Bayesian regression models for the predictive analysis. Conducting a training set selection by using samples that are closely relevant to the target population showed an improvement of prediction accuracies for most of the traits, but the optimal threshold to determine the level of relatedness cannot be determined based on our results.

The results of Scenario 3 may be limited by the sample size within each biparental family, i.e. ~20 individuals. Such a limited number of individuals in the test population may result in some alleles which are helpful in explaining the phenotypic variation in the training populations not to be included in the test population due to genetic drift, which may result in some redundant SNPs and reduce the predictive power for some families. Moreover, samples were collected from populations which had undergone significant phenotype-based selection (i.e. truncation). This reduces the phenotypic range of traits, making it difficult to obtain accurate predictions using genomic data (Table S6).

### Comparison to previous cotton genomic prediction studies

Gapare et al. (2018) used CV to evaluate prediction accuracies on 215 historical lines (a subset of the data set used in this study). They obtained prediction accuracies of 0.67 and 0.35 for LEN and STR, respectively. The prediction accuracies of these two traits in our study were considerably higher. This may be explained by the larger size of the training population in our study. Another explanation is that unlike Gapare et al. (2018) who used only genomic information to generate predictions, we have also incorporated pedigree into the models. We have shown that pedigree data provides complimentary information to the models. Thus, improved prediction accuracies can be achieved when genomics and pedigree are used in combination, as they are able to jointly describe the relatedness of individuals composited in both the training and predictive population (Velazco et al. 2019). From a breeding point of view, the training populations used in this study are predominantly recently developed CSIRO breeding lines. Given the continuity of germplasm enhancement activities in our breeding program, many elite individuals in these training populations were used as parents for new breeding crosses, from which lines in the testing populations are derived. Therefore, the relatedness between the training and testing populations is higher in this study compared to Gapare et al. (2018)’s study. This higher relatedness between the training and testing populations is likely to have influenced the improved prediction accuracies.

Another study (Islam et al. 2020) also conducted CV on 550 lines of a multiple parental cross (MAGIC) population produced in the US, and obtained maximum accuracies of 0.50 for LEN, 0.48 for UNI, 0.50 for SFI, 0.55 for STR, 0.68 for EL, and 0.35 for MIC (Fig. 2 in Islam et al. 2020). Except EL, all other traits had higher accuracies in our study. However, it is important to note that the study using a MAGIC population may not be comparable to ours due to the nature of the populations studied and the testing environments, as well as dissimilar genotyping and phenotyping methods.

### Comparison between predictive models and between CV strategies

The four Bayesian regression methods being considered in this study have different model assumptions. The BG-BLUP model assumes the genetic effects of different markers to follow a normal distribution with a common variance. Bayesian LASSO and Bayes C assume markers to follow a prior distribution with individual variance. Accordingly, BG-BLUP is most suitable to analyse a polygenic trait with all markers having small genetic effects. Alternatively, Bayesian LASSO and Bayesian C work most efficiently for oligogenic traits with small number of markers having major effects, and the rest having small effects (Li and Sillanpää 2012). The fact that these methods have similar predictive performance on our data set may indicate that the traits being analysed here have a polygenic genetic architecture so that the Bayesian LASSO and Bayes C methods do not show advantage over the BG-BLUP model.

The BART model is a non-parametric method similar to other machine learning methods which have been proposed for GP such as random forest and boosting (Li et al. 2018). BART implicitly models the genetic effects in regression decision trees. Compared to the three parametric methods, the benefit of BART is that it can also account for non-additive effects such as dominance effects and gene-gene interaction effects.

BART’s performance varies across traits and scenarios. From our analyses in Scenario 1, although BART did not provide the best accuracies among the methods for most of the traits, it indeed outperformed others for EL, MIC and LY, indicating some non-additive effects may influence those traits. However, because it is a non-parameterised method, BART cannot be used to identify the SNPs associated with those non-additive effects.

Furthermore, it must be also highlighted that adding the pedigree-based relationship matrix into the regression models improves predictions in all three scenarios. Using pedigree as a random effect is useful to account for the complex correlation structure between multiple families in the data, which cannot be fully explained by genetic data. This observation supports results published in GP research in other crop species (Velazco et al. 2019).

Another interesting technical perspective is to determine an optimal number of folds in CV (i.e. prediction Scenario 1). Our results showed that the prediction accuracies estimated by 50-fold-CV is systematically higher than the accuracies estimated by fivefold-CV, although the difference is small in magnitude. The accuracies estimated by both 50-fold-CV and fivefold-CV are all highly correlated with the square root of genomic heritability (as shown above), which indicates both approaches are suitable for evaluating prediction accuracies for GP. One major difference is however that the standard error of prediction accuracies over folds in 50-fold-CV is much larger than that of the fivefold-CV. Moreover, the computation cost of 50-fold-CV is much more expensive. Hence, the use of small number of folds in CV should be a better choice for evaluating a model’s predictability.

## Conclusion

This research presents the first GP study using samples collected from multiple years and locations from a commercial cotton breeding program. It highlights that GP models, particularly when combined with pedigree information, provide significant potential to predict accurate GEBVs (i.e. maximum prediction accuracies of 0.50–0.76, depending on the target trait). However, as no prediction model constantly outperformed all other models across the prediction scenarios and traits presented in this work, it is important to apply a set of different models to new data sets. In addition, it must be acknowledged that the circumstances where GP could be deployed in a commercial breeding program (i.e. Scenarios 2 and 3) the prediction accuracies were not consistently high. We expect that the inclusion of environmental covariates and other ‘omics’ data may help improve the accuracy of GPs. Particularly when considering complex, polygenic traits where interactions between genes and environment have significant effects on phenotype outcomes. The study can be further extended by including environmental factors such as climate variables into the statistical models; building a genotype (gene)-by-environmental interaction model to conduct prediction analyses (Jarquín et al. 2014b; Crossa et al. 2017; Rogers et al. 2021), and; the extension of prediction models to simultaneously predict multiple correlated traits (Moeinizade et al. 2020).

## Supplementary information

Supplementary tables and figures

## Data Availability

The genotype and phenotype data as well as the pedigree-based relationship matrix used in this study are available from the CSIRO Data Access Portal 10.25919/k18n-nk98.
