# Supplementary material for: Genomic prediction of cotton fibre quality and yield traits using Bayesian regression methods
Source: Heredity (Edinb). 2022 May 6;129(2):103–12. doi: 10.1038/s41437-022-00537-x (PMC9338257; doi:10.1038/s41437-022-00537-x)

Supplementary information: Genomic prediction of cotton fibre quality and yield traits using Bayesian regression methods.

Zitong Li, Shiming Liu, Warren Conaty, Qian-Hao Zhu, Philippe Moncuquet, Warwick Stiller, Iain Wilson

Supplementary Tables

Table S1. Summary of the biparental families collected since 2014.

Family ID	Experiment ID	Year	Sample size
CSX69004	1402	2014	17
CSX69017	1402	2014	22
CSX69901	1402	2014	18
CSX69009	1402	2014	12
CSX69018	1403	2014	20
CSX69014	1403	2014	11
CSX69015	1403	2014	14
CSX69008	1404	2014	13
CSX10013	1404	2014	13
CSX10015	1404	2014	17
CSX10021	1404	2014	14
CSX69022	1502	2015	19
CSX10024	1502	2015	20
CSX11015	1502	2015	21
CSX10104	1513	2015	38
CSX11102	1513	2015	41
CSX69110	1519	2015	36
CSX69111	1519	2015	18
CSX69119	1519	2015	2
CSX69120	1519	2015	4
CSX10114	1519	2015	9
CSX10221	1522	2015	18
CSX11221	1522	2015	18
CSX11813	1543	2015	15
CSX11847	1543	2015	10
CSX12809	1543	2015	21
CSX12815	1543	2015	16
CSX12816	1543	2015	8
CSX11849	1545	2015	9
CSX11850	1545	2015	11
CSX10007	1602	2016	19
CSX12017	1602	2016	18
CSX12026	1602	2016	18
CSX10014	1603	2016	19
CSX10028	1603	2016	16
CSX12028	1603	2016	17

CSX10017	1604	2016	20
CSX10018	1604	2016	16
CSX12019	1604	2016	17
CSX11207	1605	2016	10
CSX11213	1605	2016	11
CSX11219	1605	2016	10
CSX11213	1605	2016	10
CSX12011	1605	2016	15
CSX10008	1702	2017	20
CSX12008	1702	2017	21
CSX12013	1702	2017	20
CSX12018	1703	2017	18
CSX12027	1703	2017	18
CSX12029	1703	2017	17
CSX69019	1704	2017	20
CSX10011	1704	2017	21
CSX12005	1704	2017	16
CSX12010	1705	2017	14
CSX12022	1705	2017	22
CSX12022	1705	2017	20
CSX12241	1706	2017	16
CSX12243	1706	2017	10
CSX12244	1706	2017	21
CSX14504	1763	2017	29
CSX14505	1763	2017	31

Table S2. The prediction accuracies (and standard errors in brackets) of the scenario 1: five-fold Cross validation. Methods under evaluation are Bayesian G-BLUP, Bayesian LASSO, Bayes C, Bayesian additive regression tree (BART), and these four models further adding pedigree or structure information as random effects. Traits being analysed included fibre length (LEN), uniformity (UNI), short fibre index (SFI), fibre strength (STR), fibre elongation (EL), fibre micronaire (MIC), lint yield (LY) and lint percentage (LP).

	**LEN**	**UNI**	**SFI**	**STR**	**EL**	**MIC**	**LY**	**LP**
BG-BLUP	0.75 (0.05)	0.59 (0.07)	0.47 (0.06)	0.70 (0.10)	0.61 (0.06)	0.56 (0.03)	0.64 (0.05)	0.66 (0.11)
BG-BLUP + pedigree	0.77 (0.03)	0.59 (0.07)	0.47 (0.05)	0.70 (0.10)	0.62 (0.07)	0.57 (0.03)	0.65 (0.05)	0.68 (0.11)
BLASSO	0.75 (0.05)	0.59 (0.07)	0.47 (0.06)	0.70 (0.10)	0.60 (0.06)	0.56 (0.03)	0.64 (0.05)	0.66 (0.11)
BLASSO + pedigree	0.77 (0.04)	0.59 (0.07)	0.47 (0.05)	0.70 (0.10)	0.62 (0.07)	0.58 (0.02)	0.65 (0.05)	0.68 (0.11)
Bayes C	0.75 (0.05)	0.59 (0.07)	0.47 (0.06)	0.70 (010)	0.60 (0.06)	0.56 (0.03)	0.64 (0.05)	0.66 (0.11)
Bayes C + pedigree	0.77 (0.03)	0.60 (0.07)	0.48 (0.04)	0.70 (0.10)	0.62 (0.08)	0.58 (0.02)	0.64 (0.05)	0.68 (0.11)
BART	0.72 (0.05)	0.47 (0.10)	0.43 (0.07)	0.65 (0.13)	0.60 (0.10)	0.54 (0.02)	0.59 (0.06)	0.66 (0.13)
BART + pedigree	0.73 (0.06)	0.47 (0.09)	0.44 (0.07)	0.67 (0.12)	0.61 (0.09)	0.50 (0.02)	0.62 (0.05)	0.67 (0.12)

Table S3. The prediction accuracies (and standard errors in brackets) of the scenario 1: leave-one-out-fold Cross validation. Methods under evaluation are Bayesian G-BLUP, Bayesian LASSO, Bayes C, BART, and these four models further adding pedigree or structure information as random effects. Traits being analysed included fibre length (LEN), uniformity (UNI), short fibre index (SFI), fibre strength (STR), fibre elongation (EL), fibre micronaire (MIC), lint yield (LY) and lint percentage (LP).

	**LEN**	**UNI**	**SFI**	**STR**	**EL**	**MIC**	**LY**	**LP**
BG-BLUP	0.79 (0.07)	0.60 (0.16)	0.53 (0.18)	0.71 (0.11)	0.55 (0.14)	0.65 (0.11)	0.58 (0.14)	0.63 (0.15)
BG-BLUP + pedigree	0.80 (0.07)	0.60 (0.17)	0.51 (0.18)	0.71 (0.11)	0.58 (0.14)	0.67 (0.09)	0.60 (0.16)	0.64 (0.16)
BLASSO	0.79 (0.07)	0.60 (0.16)	0.53 (0.18)	0.71 (0.11)	0.55 (0.14)	0.66 (0.10)	0.58 (0.15)	0.63 (0.14)
BLASSO + pedigree	0.80 (0.07)	0.60 (0.17)	0.51 (0.18)	0.72 (0.11)	0.58 (0.14)	0.67 (0.09)	0.60 (0.16)	0.64 (0.16)
Bayes C	0.80 (0.07)	0.60 (0.17)	0.53 (0.17)	0.71 (0.12)	0.55 (0.14)	0.66 (0.10)	0.58 (0.14)	0.63 (0.16)
Bayes C + pedigree	0.81 (0.07)	0.60 (0.16)	0.52 (0.18)	0.71 (0.11)	0.57 (0.15)	0.67 (0.09)	0.60 (0.16)	0.64 (0.18)
BART	0.78 (0.07)	0.49 (0.16)	0.53 (0.17)	0.67 (0.14)	0.58 (0.14)	0.65 (0.12)	0.60 (0.14)	0.59 (0.13)
BART + pedigree	0.79 (0.09)	0.50	0.54	0.70 (0.12)	0.60 (0.13)	0.65 (0.10)	0.61	0.62

Table S4. The prediction accuracies of the scenario 2: the lines phenotyped at seasons 1993-2016 were used as the training population, and the data collected in the 2017/18 season were used as the test population. Methods under evaluation are Bayesian G-BLUP, Bayesian LASSO and Bayes C, and the three models further adding pedigree or structure information as random effects. Traits being analysed included fibre length (LEN), uniformity (UNI), short fibre index (SFI), fibre strength (STR), fibre elongation (EL), fibre micronaire (MIC), lint yield (LY) and lint percentage (LP).

	**LEN**	**UNI**	**SFI**	**STR**	**EL**	**MIC**	**LY**	**LP**
BG-BLUP	0.41	0.14	0.18	0.35	0.43	0.23	0.17	0.30
BG-BLUP + pedigree	0.42	0.08	0.14	0.38	0.41	0.19	0.18	0.36
BLASSO	0.39	0.11	0.19	0.36	0.43	0.25	0.14	0.31
BLASSO + pedigree	0.42	0.10	0.20	0.37	0.43	0.23	0.15	0.32
Bayes C	0.42	0.09	0.20	0.36	0.43	0.22	0.17	0.31
Bayes C + pedigree	0.42	0.11	0.18	0.38	0.41	0.19	0.14	0.35
BART	0.42	0.10	0.16	0.35	0.48	0.28	0.25	0.32
BART+pedigree	0.42	0.11	0.15	0.38	0.46	0.23	0.25	0.33

Table S5. The prediction accuracies of the scenario 3. This approach used each biparental family from season 2017/18 as the separate test population. The training population was either all the lines phenotyped before 2017, or the families phenotyped before 2017 which are closely relevant to the target population (i.e. the related coefficient no less than 0.125 or 0.25). Numbers highlighted in bold represent the highest prediction accuracies among different approaches.

Trait/test population	All accessions phenotyped before 2017 as training data		Relationship coefficient≥0.125			Relationship coefficient≥0.25		
	BLASSO	BLASSO + pedigree	Training sample size	BLASSO	BLASSO + pedigree	Training sample size	BLASSO	BLASSO + pedigree
LEN								
CSX10008	0.55	**0.59**	686	0.58	0.57	356	0.40	0.38
CSX12008	0.14	0.26	667	0.31	**0.37**	256	0.25	0.28
CSX12013	0.00	**0.17**	667	0.14	**0.17**	256	0.00	0.00
CSX12018	0.00	0.00	707	0.00	0.00	483	0.00	0.00
CSX12027	0.00	0.00	712	0.00	0.00	481	0.00	**0.30**
CSX12029	**0.59**	0.51	728	0.41	0.39	450	0.32	0.41
CSX69019	0.13	0.26	703	0.06	0.17	264	**0.30**	0.14
CSX10011	0.49	0.47	712	0.26	0.24	331	0.12	0.09
CSX12010	0.15	**0.26**	681	0.16	0.17	327	0.11	0.19
Mean	0.23	**0.28**		0.21	0.23		0.17	0.20
SD	0.25	0.21		0.20	0.19		0.15	0.15
UNI								
CSX10008	0.03	**0.05**	686	0.04	0.03	356	0	0.01
CSX12008	0.50	0.56	667	0.58	**0.61**	256	0.66	0.65
CSX12013	**0.22**	0.11	667	0	0	256	0	0
CSX12018	0.33	**0.44**	707	0.39	0.29	483	0.34	0.27
CSX12027	0	0	712	0.07	**0.17**	481	0.05	0.14
CSX12029	**0.25**	0.19	728	0.23	0.23	450	0.16	0.17
CSX69019	0.30	0.25	703	0.30	0.32	264	0.31	**0.35**
CSX10011	0.18	0.15	712	0.22	0.20	331	**0.27**	0.22
CSX12010	**0.46**	0.55	681	0.48	0.50	327	**0.63**	0.61
**Mean**	**0.25**	**0.26**		**0.27**	**0.26**		**0.27**	**0.27**
**SD**	**0.17**	**0.21**		**0.20**	**0.20**		**0.25**	**0.23**
SFI								
CSX10008	0.20	0.33	686	0.34	**0.36**	356	0.31	0.30
CSX12008	0.40	0.41	667	0.43	0.45	256	0.39	**0.46**
CSX12013	0	**0.08**	667	0	0	256	0	0
CSX12018	0	0	707	0	0	483	0	0
CSX12027	0	0	712	0	0	481	0	0
CSX12029	0.19	0.23	728	**0.24**	0.23	450	0.22	0.21
CSX69019	0.02	0.13	703	0.13	0.16	264	**0.24**	**0.24**
CSX10011	0.10	0.04	712	0	0	331	**0.17**	0.16

CSX12010	0.32	0.41	681	0.40	**0.42**	327	0.33	0.37
**Mean**	**0.14**	**0.18**		**0.17**	**0.18**		**0.18**	**0.19**
**SD**	**0.15**	**0.17**		**0.18**	**0.19**		**0.15**	**0.17**
**STR**								
CSX10008	0.49	0.50	686	0.31	0.32	356	**0.54**	0.50
CSX12008	0.38	0.38	667	0.42	**0.44**	256	0.43	0.43
CSX12013	0.60	**0.62**	667	0.58	0.57	256	0.39	0.39
CSX12018	0.00	0.00	707	0.06	0.16	483	**0.29**	0.25
CSX12027	0.41	0.42	712	0.36	0.34	481	**0.45**	0.42
CSX12029	0.31	**0.38**	728	0.26	0.27	450	0.28	0.29
CSX69019	0.40	0.46	703	0.33	0.27	264	0.58	**0.59**
CSX10011	0.59	0.63	712	0.66	0.70	331	0.71	**0.73**
CSX12010	0.44	0.47	681	0.56	0.59	327	0.58	**0.60**
**Mean**	**0.40**	**0.43**		**0.39**	**0.41**		**0.47**	**0.47**
**SD**	**0.18**	**0.18**		**0.19**	**0.18**		**0.14**	**0.15**
**EL**								
CSX10008	**0.47**	0.43	686	0.33	0.33	356	0.29	0.28
CSX12008	**0.43**	0.29	667	0.10	0.13	256	0.17	0.19
CSX12013	0.33	**0.34**	667	0	0	256	0	0
CSX12018	0.06	**0.12**	707	0	0	483	0	0
CSX12027	0	0.02	712	0	0	481	**0.08**	0.06
CSX12029	0.48	0.36	728	**0.57**	0.54	450	0.44	0.38
CSX69019	0.46	0.48	703	0.53	**0.54**	264	0.28	0.35
CSX10011	0.27	**0.34**	712	0.11	0.13	331	0.07	0.13
CSX12010	0.23	0.25	681	0.26	0.26	327	**0.38**	0.37
**Mean**	**0.30**	**0.29**		**0.21**	**0.21**		**0.19**	**0.20**
**SD**	**0.18**	**0.15**		**0.22**	**0.22**		**0.16**	**0.16**
**MIC**								
CSX10008	0.12	0.20	686	0.34	0.35	356	**0.37**	0.35
CSX12008	0.44	**0.46**	667	0.44	**0.46**	256	0.23	0.25
CSX12013	0	**0.01**	667	0	0	256	0	0
CSX12018	0.28	0.42	707	0.44	0.49	483	0.49	**0.50**
CSX12027	0.41	0.43	712	0.56	0.57	481	0.62	**0.63**
CSX12029	0	0	728	0	0	450	0	0
CSX69019	0.05	0.05	703	0	0	264	0.1	**0.08**
CSX10011	0.40	0.42	712	0.41	**0.45**	331	0.26	0.28
CSX12010	0.31	0.30	681	0.47	0.49	327	0.64	**0.62**
**Mean**	**0.22**	**0.25**		**0.30**	**0.31**		**0.30**	**0.30**
**SD**	**0.17**	**0.18**		**0.21**	**0.23**		**0.23**	**0.23**
**LY**								
CSX10008	0.33	0.27	686	**0.32**	0.30	356	0.21	0.21
CSX12008	0.02	0.17	667	0.12	0.18	256	0.25	**0.29**
CSX12013	0.41	0.40	667	0.42	0.40	256	0.48	**0.50**
CSX12018	0	0.11	707	0.34	**0.35**	483	0.32	0.33
CSX12027	**0.22**	0.19	712	0.07	0.06	481	0.17	0.13

CSX12029	**0.18**	0.17	728	**0.18**	0.12	450	0.16	0.11
CSX69019	0.02	0	703	0	0	264	0.02	**0.05**
CSX10011	0	0	712	0.02	0.04	331	**0.40**	0.38
CSX12010	0.32	0.37	681	**0.50**	0.42	327	0.33	0.33
**Mean**	**0.13**	**0.19**		**0.22**	**0.21**		**0.26**	**0.26**
**SD**	**0.16**	**0.14**		**0.18**	**0.16**		**0.14**	**0.15**
**LP**								
CSX10008	0.58	0.61	686	0.62	**0.67**	356	0.49	0.52
CSX12008	0.27	0.37	667	0.33	**0.40**	256	0.22	0.26
CSX12013	0.61	0.60	667	**0.62**	0.59	256	0.42	0.42
CSX12018	0.27	0.27	707	0.28	0.26	483	0.42	**0.45**
CSX12027	0.32	0.51	712	0.52	**0.58**	481	0.51	0.43
CSX12029	0.17	0.22	728	0.28	0.26	450	0.36	**0.36**
CSX69019	0.73	0.70	703	**0.76**	0.70	264	0.68	0.58
CSX10011	0.44	0.49	712	**0.53**	0.48	331	0.17	0.16
CSX12010	0	0.06	681	**0.25**	0.15	327	0	0
**Mean**	**0.38**	**0.43**		**0.47**	**0.45**		**0.36**	**0.35**
**SD**	**0.23**	**0.21**		**0.19**	**0.20**		**0.20**	**0.18**

Table S6. The standard deviation (SD) of fibre length (LEN) and strength (STR) (after standardization of the phenotypes by checks in each trial) in each biparental family collected in the season 2017/18. The SDs of LEN is systematically lower than the ones of STR, which may partially explain why the prediction accuracies in LEN is considerably lower than the STR in Scenario 3.

Crosses	Number of lines	LEN	STR
CSX10008	20	0.028	0.035
CSX12008	21	0.024	0.028
CSX12013	20	0.023	0.038
CSX12018	18	0.028	0.026
CSX12027	18	0.015	0.028
CSX12029	17	0.032	0.032
CSX69019	20	0.024	0.040
CSX10011	21	0.020	0.046
CSX12010	14	0.034	0.038

Supplementary Figures

Figure S1. The pedigree chart of 1385 lines being used in genomic prediction analysis. In the figure, Circles in grey, dark blue, light blue, light Green, and dark green represent lines and/or families collected from the pre-2014 trials, year 2014, 2015, 2016 and 2017, respectively.

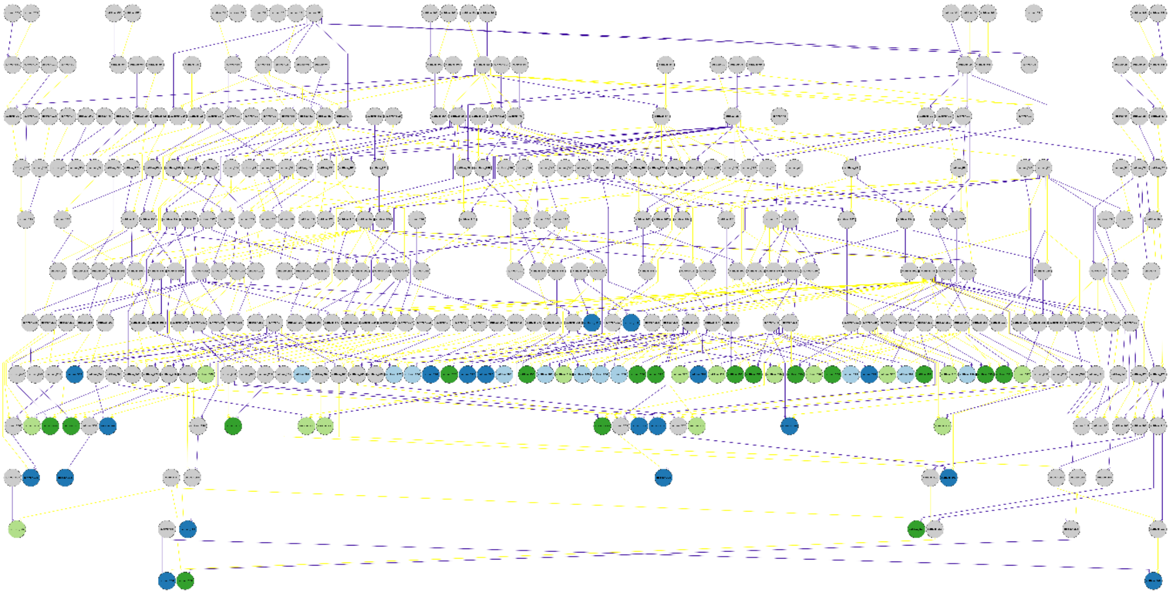

Figure S2. Principal component analysis on the genotype data of 1385 lines.

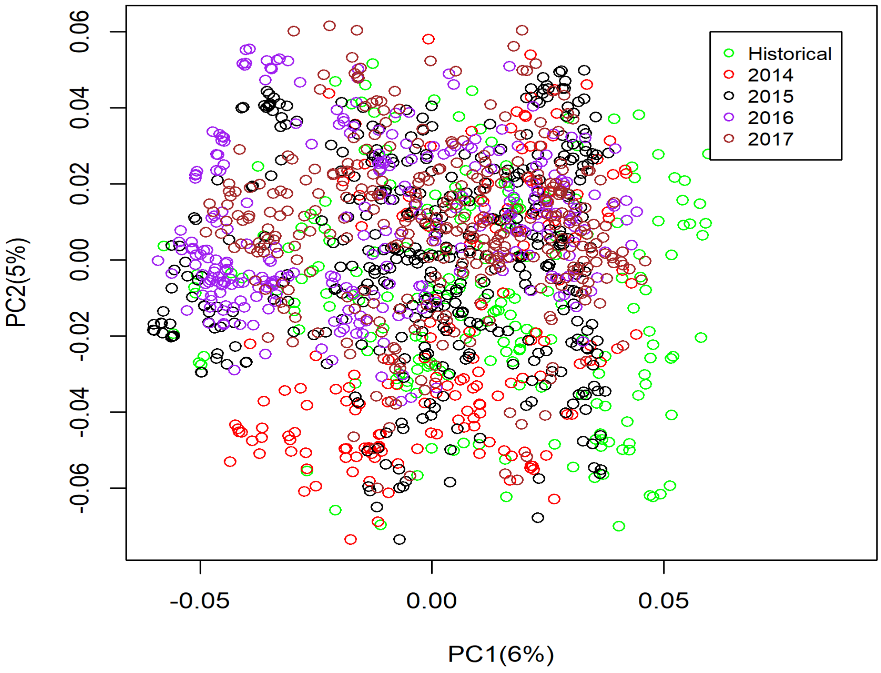

Supplement: Supplementary file 1 — Supplementary tables and figures [file 41437_2022_537_MOESM1_ESM.pdf]
